# 1541. HIV Risk Assessment in Boston Community Health Center Dental Clinics

**DOI:** 10.1093/ofid/ofad500.1376

**Published:** 2023-11-27

**Authors:** Daryl Konstandt, Pegah Mohammah Alizadeh Chafjiri, Eric Bolter, Elisabeth Collins, Sara Lodi, Michelle Henshaw, Lauri Bazerman, Janice Cho, Caitlin Coleman, Mina Paul, Elizabeth Powell, Curt Beckwith

**Affiliations:** Boston University, Boston, Massachusetts; Boston University, Boston, Massachusetts; Boston University, Boston, Massachusetts; Boston University, Boston, Massachusetts; Boston University School of Public Health, Boston, Massachusetts; Boston University, Boston, Massachusetts; The Miriam Hospital, Providence, Rhode Island; Boston University, Boston, Massachusetts; Harbor Health Services, Inc., Boston, Massachusetts; Boston University, Boston, Massachusetts; Boston University, Boston, Massachusetts; Alpert Medical School of Brown University, Providence, Rhode Island

## Abstract

**Background:**

Suffolk County (Boston), Massachusetts is identified by the CDC as a geographic hotspot for new HIV infections. The Ending the HIV Epidemic Initiative (EHE), implemented by the Department of Health and Human Services, aims to decrease new HIV infections by 90% in the next decade. Although HIV treatment radically improves lives, individuals must know their status to receive treatment. Fourteen percent of persons with HIV are unaware of their diagnosis and 23% of dental patients do not access primary medical care. Therefore, community health center dentists are uniquely positioned to contribute to the “Diagnose” pillar of EHE. Dental clinics can provide new opportunities for HIV testing for persons at increased risk for HIV within EHE designated jurisdictions. The purpose of this study was to assess HIV risk factors [sexually active men who have sex with men (MSM), multiple sexual partners, condomless sex, and intravenous drug use (IDU)] among community health center dental patients in the Boston area.

**Methods:**

An anonymous, voluntary, 25 question survey was disseminated in English, Spanish and Haitian Creole within 5 community health center dental clinics in Boston. The data were compiled and analyzed with descriptive statistics using REDCap survey software and EXCEL.

**Results:**

Of the 299 dental patients who completed the survey, the majority were female (55%), black (54%), non-Hispanic (52%) and 22-54 years old (56%). 24% of respondents reported at least one risk factor for HIV within the past year including 53 individuals with multiple sexual partners, 18 sexually active MSM and 3 IDU in the past year. Of the 188 persons that had sexual contacts in the past 12 months, 37% never used a condom. Thirty-four percent report never having an HIV test.

**Conclusion:**

One-quarter of dental clinic patients who completed the survey reported HIV risk factors, making community health center dental clinics an underutilized venue for HIV testing and prevention interventions. The goals of EHE require the implementation of HIV testing in new settings to reach persons who are missed by traditional HIV testing sites. Dental providers represent an important, yet untapped, workforce that can contribute to EHE.

**Disclosures:**

**All Authors**: No reported disclosures

